# Histone deacetylases (HDACs) in XPC gene silencing and bladder cancer

**DOI:** 10.1186/1756-8722-4-17

**Published:** 2011-04-20

**Authors:** Xiaoxin S Xu, Le Wang, Judith Abrams, Gan Wang

**Affiliations:** 1 Institute of Environmental Health Sciences, Wayne State University, 259 Mack Avenue, Detroit, MI 48201, USA; 2 Karmanos Cancer Institute, Wayne State University, 4100 John R Street, Detroit, MI 48201 USA

## Abstract

Bladder cancer is one of the most common malignancies and causes hundreds of thousands of deaths worldwide each year. Bladder cancer is strongly associated with exposure to environmental carcinogens. It is believed that DNA damage generated by environmental carcinogens and their metabolites causes development of bladder cancer. Nucleotide excision repair (NER) is the major DNA repair pathway for repairing bulk DNA damage generated by most environmental carcinogens, and XPC is a DNA damage recognition protein required for initiation of the NER process. Recent studies demonstrate reduced levels of XPC protein in tumors for a majority of bladder cancer patients. In this work we investigated the role of histone deacetylases (HDACs) in XPC gene silencing and bladder cancer development. The results of our HDAC inhibition study revealed that the treatment of HTB4 and HTB9 bladder cancer cells with the HDAC inhibitor valproic acid (VPA) caused an increase in transcription of the XPC gene in these cells. The results of our chromatin immunoprecipitation (ChIP) studies indicated that the VPA treatment caused increased binding of both CREB1 and Sp1 transcription factors at the promoter region of the XPC gene for both HTB4 and HTB9 cells. The results of our immunohistochemistry (IHC) staining studies further revealed a strong correlation between the over-expression of HDAC4 and increased bladder cancer occurrence (*p *< 0.001) as well as a marginal significance of increasing incidence of HDAC4 positivity seen with an increase in severity of bladder cancer (*p *= 0.08). In addition, the results of our caspase 3 activation studies demonstrated that prior treatment with VPA increased the anticancer drug cisplatin-induced activation of caspase 3 in both HTB4 and HTB9 cells. All of these results suggest that the HDACs negatively regulate transcription of the XPC gene in bladder cancer cells and contribute to the severity of bladder tumors.

## Introduction

Bladder cancer is one of the most common malignancies. Worldwide, more than 350,000 new cases of bladder cancer are diagnosed each year with over 145,000 deaths resulting from the disease [1]. Bladder cancer is strongly associated with exposure to environmental factors. Cigarette smoking is the single most important environmental factor in causing bladder cancer [2]. Exposure to other environmental factors, especially polycyclic aromatic amines, such as aniline, benzidine, and turoline, is also closely correlated with bladder cancer risk [2]. The mechanism by which the exposure to environmental factors causes development of bladder cancer is unknown. It is believed that the exposure to the environment makes the bladder tissue more susceptible to environmental carcinogens and the DNA damage generated by these carcinogens and/or their metabolites causes initiation and progression of bladder cancer.

Nucleotide excision repair (NER) is the major DNA repair pathway in repairing bulky DNA damage generated by most environmental carcinogens, including DNA damage generated by cigarette smoking [3-5]. The NER pathway can be further distinguished into the transcription-coupled NER (TCR) and global genome NER (GGR) sub-pathways. The TCR pathway quickly repairs DNA damage in highly transcribed DNA sequences, whereas the GGR pathway repairs DNA damage throughout the entire genome, but at a dramatically decreased rate [6,7]. In TCR, DNA damage is recognized by a stalled transcription event [8,9], whereas in GGR, DNA damage is recognized by XPC, a DNA damage recognition protein [10,11]. The DNA damage recognition signal further recruits several important NER components, including XPA, RPA, TFIIH, XPG, and XPF-ERCC1, to the damage site [4]. The dual incisions made by XPG [12] and XPF-ERCC1 [13,14] generates a 22-24nt single-stranded gap. The DNA polymerases (pol δ and ε) fill the gap using the complementary DNA strand as a template and DNA ligase seals the flanking gaps to complete the DNA repair process [15].

Beyond its role in DNA repair, the DNA damage recognition signal of XPC protein is also required for many DNA damage-induced cellular responses, including cell cycle checkpoint regulation and apoptosis [16]. Activation of p53, a key DNA damage signaling-mediator [4], is involved in the XPC protein DNA damage recognition-induced signaling process [16]. The protein-protein interactions of the XPC protein with other NER components, most notably TFIIH [17-19], seem to play a critical role in the DNA damage-mediated signal transduction process. The active p53 protein further induces transcription of important DNA damage-responsive genes to result in relevant cellular responses. Therefore, the presence of a functional XPC protein is essential not only for DNA repair, but also for DNA damage-mediated signal transduction, which results in restoration of the disrupted cellular functions or elimination of the severely damaged cells.

Deficiency or attenuation of the XPC protein has been strongly associated with high incidence of cancer. The patients of xeroderma pigmentosum (XP), including XPC patients, display an over 1000-fold increase in skin cancer incidence [5,20,21]. The XPC patients also display high incidences of lung, liver, and colon cancer [5]. Transgenic animal studies reveal that XPC gene knockout mice (XPC^-/-^) develop significantly higher levels of skin, liver, and lung tumors than their wild type (XPC^+/+^) or XPC heterozygous (XPC^+/-^) littermates when exposed to chemical carcinogens [22-27]. The results obtained from others and our recent studies reveal reduced levels of XPC protein in the tumors for a majority of bladder and lung cancer patients [27-29]. All of these results suggest that the presence of a functional XPC protein is essential in protecting cells against environmental carcinogen-caused cancer development, and XPC protein attenuation and its deficiency contributes to cancer development, especially for cancers strongly associated with environmental factors such as lung and bladder cancer. In addition, reduced levels of XPC protein may also be a contributing factor in tumor cell resistance to many commonly used DNA-damaging anticancer drugs because of the role of the XPC protein in initiating important cellular responses such as apoptosis following the treatment with these drugs.

The mechanism that leads to reduced levels of XPC protein in the tumors of bladder cancer patients is unknown. The knowledge obtained from recent epigenetic studies suggests that epigenetic regulation may play an important role in this aspect [30-35]. The epigenetic regulation involves several different mechanisms, including DNA methylation, histone acetylation/deacetylation, and microRNA (miRNA). In regards to histone acetylation/deacetylation, it is widely known that the acetylation status of histones significantly affects transcription of target genes [36]. The binding of acetylated histones at the promoter region of target genes leads to a more opened chromatin structure, which enhances transcription of the target gene. In contrast, the binding of deacetylated histones at the promoter region causes a more closed DNA structure, which causes silencing of the target gene. Deacetylation of the histones occurs through histone deacetylases (HDACs), a super family of proteins [37]. Abnormal levels of deacetylases have been reported in many types of cancer, which suggests a possible role of HDACs in the disease process [37,38].

In this study, we focused on determining the role of histone deacetylases (HDACs) in XPC gene silencing and bladder cancer development. Using HTB4 (T24) and HTB9 bladder carcinoma cells, the results of our HDAC inhibitor studies demonstrated that treatment with a HDAC inhibitor, valproic acid (VPA), caused increased transcription of the XPC gene in these cells. The results obtained from our chromatin immunoprecipitation (ChIP) studies revealed that the treatment of VPA enhanced the binding of transcription factors CREB-1 and Sp1 at the promoter region of the XPC gene in both HTB4 and HTB9 cells. The results obtained from our immunohistochemistry (IHC) staining studies further revealed a strong correlation between the over-expression of HDAC4 and the occurrence of bladder transitional cell carcinomas (*p *< 0.001) as well as a marginal significance between the over-expression of HDAC4 and the severity of the bladder tumors (*p *= 0.08). In addition, the results of our caspase 3 activation studies demonstrated that the prior treatment with VPA enhanced the anticancer drug cisplatin-induced activation of caspase 3 in both HTB4 and HTB9 cells. All of these results suggest that over-expression of the HDAC4 contributes to the XPC gene silencing and the development of bladder carcinomas, and inhibiting the HDAC activities with the HDAC inhibitor VPA sensitizes the bladder carcinoma cells to anticancer drug cisplatin. These results provide an important mechanism for the XPC gene silencing in bladder cancer cells and suggest an important mechanism in bladder cancer development. In addition, the results obtained from this study also suggest that inhibiting HDAC activity with HDAC inhibitor may greatly benefit the bladder cancer treatment through its sensitization of bladder cancer cells to many DNA-damaging anticancer drugs, such as cisplatin.

## Materials and methods

### Cell lines and Oligonucleotides

The HTB4 (T24), HTB9, HTB2, HTB3, HTB5, HT1197, and HT1376 bladder cancer cells were purchased from American Type Culture Collection (ATCC) (Rockville, MD). The GM00637 human fibroblast cells were purchased from the Coriell Institute for Medical Research (Camden, NJ). The HTB2 and HTB4 cells were cultured in a McCoy's 5A medium supplemented with 10% FBS at 37°C with 5% CO_2_. The HTB9 cells were cultured in RPMI1640 medium supplemented with 1× non-essential amino acids (NEAA) and 10% FBS at 37°C with 5% CO_2_. The HTB3, HTB5, HT1197, and HT1376 bladder cancer cells were cultured in minimal essential medium (MEM) supplemented with 10% FBS and 1× NEAA at 37°C with 5% CO_2_. The GM00637 cells were cultured in MEM supplemented with 10% FBS, 2× essential amino acids (EAA), 2x NEAA, and 2x vitamins (Vt) at 37°C with 5% CO_2_.

The oligonucleotides used in this study are listed in Table 1 and were synthesized by Retrogen, Inc. (San Diego, CA). The primers used for determining the level of XPC mRNA by real time PCR were designed to bind to the XPC mRNA sequence at exon 5 and exon 6 thus amplifying a 120 bp DNA fragment. The primers used for determining the level of XPA mRNA by real time PCR were designed to bind to the XPA mRNA at exon 3 and exon 4 in order to amplify a 110 bp DNA fragment. The primers used for detection of the immuno-precipitation XPC gene promoter sequence were designed to bind to the XPC gene 5' regulatory region sequence at the -95 to -75 region and the +80 to +50 region to amplify a 175 bp DNA fragment.

**Table 1:** Oligonucleotides used in the study.

Name of oligonucleotide	Sequences of the oligonucleotide
1. Primers used for the real time PCR study	
XPC primer 1	5'-GTGACCTCAAGAAGGCACAC-3'
XPC primer 2	5'-CTCACGTCACCCAGCACAGG-3'
XPA primer 1	5'-CTGCGGCTACTGGAGGCATGG-3'
XPA primer 2	5'-CCATAACAGGTCCTGGTTGATG-3'
2. Primers used for amplifying the XPC gene 5' regulatory region in the IP study	
XPC IP primer 1	5'-CGTGGCCAAGCGCACCGCCTC-3'
XPC IP primer 2	5'-GGCCTTGCTCTTGGCCTTG-3'

### VPA treatment

The VPA was purchased from Sigma Corp. (St. Louis, MO). The HTB4 and HTB9 cells were seeded onto 100 mm cell culture dishes at a density of 1 × 10^6 ^cells/dish and incubated at 37°C overnight. The VPA was added to the cell culture medium to a final concentration of 5 mM. The cells were cultured in the VPA-containing medium for 48 hours and then used for further studies.

### Real time quantitative PCR assay

Total RNA was isolated from both untreated and VPA-treated HTB4 and HTB9 bladder cancer cells using an RNeasy mini isolation kit (Qiagen). A reverse transcription-based quantitative PCR (real time PCR) was then performed to determine the mRNA levels of both *xpc *and *xpa *genes from each RNA sample using a Sybr green-based DNA quantification method (Applied Biosystems, Foster City, CA). The mRNA level of the *β-actin *gene was also determined for each RNA sample by using the real time PCR. The reverse transcription assay was carried out using 2 μg of total RNA utilizing the protocol suggested by the manufacturer (Applied Biosystems). The PCR procedure was performed using Taq-Man Universal PCR master mix with 100 ng cDNA in a total volume of 20 μl. The PCR assays were completed using the ABI prism 7500 Fast PCR system with the following conditions: 2 min at 94°C, followed by 40 cycles of 15 seconds at 95°C, 30 seconds at 56°C, and 60 seconds at 72°C. The real time PCR data was analyzed using a comparative cycle threshold (C_t_) method. Relative quantification was performed to determine gene expression between untreated and VPA-treated cells. The *actin *gene was used as an internal control for normalization. Relative transcriptions of the XPC and XPA mRNAs were calculated as 2^-ΔΔCt ^where ΔC_t _was calculated by subtracting the average actin gene C_t _from the average XPC or XPA gene C_t _value in the same cell line. The ΔΔC_t _was obtained by the ΔC_t _of the VPA-treated cells subtracted from the ΔC_t _of the untreated cells.

### Western blot hybridization and quantification of the protein

Cells were harvested and lysed in RIPA cell lysis buffer (1xPBS, 1% NP40, 0.5% deoxycholic acid, 0.1% SDS). The cell lysates (30 μg total protein) were analyzed by SDS-PAGE using a 10% gel. The proteins were transferred to a PVDF membrane and hybridized with the indicated antibodies for detection of the desired target proteins. The same membrane was then soaked in a stripping solution (62.5 mM Tris, pH 6.8, 2% SDS, 0.7% 2-mercaptoethanol) at 50°C for 30 min and then hybridized with a β-actin antibody (Oncogene, Cambridge, MA) to determine the level of β-actin in each sample. Quantification of the western results was performed using a Kodak Image Station 440CF system and the level of the target protein in each cell lysate was expressed as a relative level to that of β-actin in the same cell lysate. The level of XPC protein in the VPA-treated cells was calculated as a percentage compared to that of the XPC protein in the untreated cells. The statistical analysis of the western data was done using GraphPad PRISM 4.0 software.

### Chromatin immunoprecipitation (ChIP)

The cells were harvested and washed in 1xPBS buffer once. The cells were then resuspended into 1xPBS buffer containing 1% formaldehyde and incubated at 37°C for 15 minutes. The cells were collected and washed three times with 1xPBS buffer. The cells were then resuspended into SDS lysis buffer (1 × 10^6 ^cells/200 μl) and incubated on ice for 10 minutes. The cells were sonicated in order to shear the genomic DNA to lengths of 200-1000 bp. The cell lysates were centrifuged at 4°C for 10 minutes and the supernatants were collected. For the ChIP assay, cell lysate (200 μl) was diluted at a ratio of 1:10 in the ChIP dilution buffer (0.01% SDS, 1.1% Triton X-100, 1.2 mM EDTA, 16.7 mM Tris-HCl, pH8.1, 167 mM NaCl) and incubated with either Protein A-conjugated agarose beads (for Sp1) or Protein G-conjugated agarose beads (for CREB1) at 4°C for 60 minutes. The cell lysates were centrifuged at 4°C for 5 minutes to remove the agarose beads. The cell lysates were then incubated with 2 μg of CREB1 antibody (X-12 from Santa Cruz) or Sp1 antibody (H-225 from Santa Cruz) at 4°C overnight using a rotating mixer. The Protein A-conjugated agarose beads (for Sp1) or Protein G-conjugated agarose beads (for CREB1) were then added and the reactants were incubated at 4°C for 2 hours with a rotating mixer. The beads were collected and washed three time in 1xPBS buffer and three times in ChIP washing buffer (0.1%SDS, 1% Triton X-100, 2 mM EDTA, 20 mM Tris-HCl, pH8.1, 150 mM NaCl). Half of the beads were analyzed by western blot to determine the amount of the CREB1 or Sp1 proteins precipitated by the ChIP protocol. The remainder of the beads were resuspended into 200 μl of DNA elution buffer (0.1M Na_2_CO_3_, 1% SDS, 200 mM NaCl) and incubated at 65°C for 6 hours to reverse the protein-DNA cross-links. The DNA was recovered by phenol/chloroform extraction and ethanol precipitation. The relative level of XPC gene promoter region DNA co-precipitated with the beads was determined by a quantitative PCR (qPCR) protocol using the Applied Biosystems' Fast 7500 Real Time PCR system (Applied Biosystems, Foster City, CA). The level of the XPC gene promoter region DNA co-precipitated with the CREB1 or Sp1 in the untreated cells was accounted as 100% and the level of the XPC gene promoter region DNA co-precipitated with the beads in the VPA-treated cells was calculated as a fold change relative to that of the untreated cells.

### Immunohistochemistry (IHC) staining

The bladder tumor tissue arrays BL208, BL2081 and BL2082 were purchased from US BioMax Inc. (Rockville, MD) and were used in the IHC staining study. The formalin-fixed paraffin-embedded (FFPE) bladder tumor tissue array slides were first deparaffinized in 100% xylenes; the slides were then hydrated through a series of graded alcohols (100%, 95%, 80%, 70%, and 30%) for 5 minutes each. The slides were washed once in H_2_O for 5 minutes. The slides were then incubated in 10 mM sodium citrate buffer (pH6.0) for 15 minutes at 95°C to unmask the antigen. The bladder tumor tissue array slides were then incubated in 1% hydrogen peroxide at room temperature for 10 minutes to quench endogenous peroxidase activity. The slides were incubated in 1.5% normal blocking serum in 1xPBS for 1 hour and then incubated with the primary antibody at 1:100 dilution in 1xPBS for 30 minutes. The slides were washed in 1xPBS three times and then incubated with a biotin-conjugated secondary antibody (Santa Cruz) at room temperature for 30 minutes. The slides were then washed three times in 1xPBS and incubated with an avidin-biotin enzyme reagent (Santa Cruz) for 30 minutes. The slides were incubated in peroxidase substrate (Santa Cruz) for 1 to 10 minutes until the desired stain intensity developed. The slides were counterstained in Gill's formulation #2 hematoxylin (Santa Cruz) for 10 seconds and then washed in deionized H_2_O with several H_2_O changes. The slides were dehydrated through graded alcohols (30 - 100%) and xylenes and mounted with glass coverslips using a Clarion permanent mounting medium (Santa Cruz, CA). The HDAC-positive cells were determined using light microscopy. Two hundred cells were counted from each tissue specimen. A HDAC-negative tissue specimen was established if >20% of the counted cells were HDAC-positive cells and a HDAC-positive tissue specimen was established if <20% of the counted cells were HDAC-positive cells.

### Caspase-3 assay

The caspase-3 activity was measured using a protocol described previously [39,40]. Essentially, the cells were harvested 40 hours after the cisplatin treatment and lysed in insect cell lysis buffer (BD Biosciences). The protein concentrations of the cell lysates were determined. The caspase-3 assay was carried out in a 96-well plate using fluorogenic Ac-DEVD-AMC as a substrate (BD Biosciences). Caspase-3 activity was determined by a spectrafluorometer (Molecular Devices) for detection of free AMC released from the substrate during a 15-minute incubation period at 37°C with an excitation wavelength of 380 nm and an emission wavelength of 430-460 nm. Caspase-3 activity was measured as nanomole of AMC/min/mg protein

### Statistical analysis

Results were expressed as the mean + standard deviation (S.D.). Statistically significant differences were determined using a one-factor analysis of variance with *p *< 0.01. The data was obtained from at least three independent experiments.

## Results

### Induced transcription of XPC gene in the VPA-treated HTB4 and HTB9 bladder cancer cells

In order to determine the role that the HDACs may play in XPC gene silencing and bladder cancer development, we first determined the effect of HDAC inhibitor treatment on activation of XPC gene transcription in HTB4 and HTB9 bladder cancer cells. Both HTB4 and HTB9 cancer cells were treated with the HDAC inhibitor of VPA (5 mM) for 48 hours and the total RNA was isolated. Total RNA was also isolated from the untreated HTB4 and HTB9 cells. A reverse transcription-based quantitative PCR (real time PCR) was performed to determine the level of the XPC mRNA in each RNA sample (Table 2). The level of XPA mRNA was also determined for each RNA sample in the study. The XPA protein is an important NER component but the level of XPA mRNA was not affected by the DNA damaging treatment [16]. The level of β-actin mRNA was determined for each RNA sample as an internal control. The level of the XPC mRNA increased 2.8 ± 0.4 and 2.4 ± 0.3 fold in the HTB4 and HTB9 cells respectively with the VPA treatment (Table 2). In contrast, the level of the XPA mRNA was not significantly altered in these cells following the VPA treatment (Table 2). These results suggest that the HDACs indeed play an important role in XPC gene silencing for both HTB4 and HTB9 bladder cancer cells, and treatment with the VPA HDAC inhibitor causes activation of the XPC transcription in both bladder cancer cell lines.

**Table 2:** The effect of valproic acid (VPA) treatment on transcription of XPC and XPA genes in both HTB4 and HTB9 bladder cancer cells.

Genes	HTB4		HTB5	
	
	No treatment	VPA treatment	No treatment	VPA treatment
XPC mRNA	1	2.8 ± 0.4	1	2.4 ± 0.3

XPA mRNA	1	1.1 ± 0.1	1	0.9 ± 0.1

### The VPA treatment caused enhanced binding of the CREB1 and Sp1 transcription factors at the promoter region of the endogenous XPC gene in both HTB4 and HTB9 bladder cancer cells

**Figure 1:**
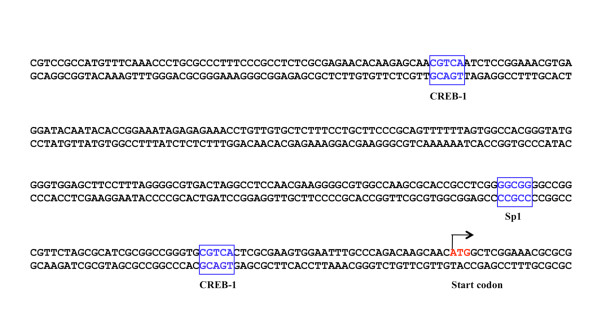
**Diagram of the promoter region structure of the XPC gene**. The consensus sequences of transcription factors CREB-1 and Sp1 were highlighted in the box. The start codon of the XPC gene is labeled in red.

**Figure 2:**
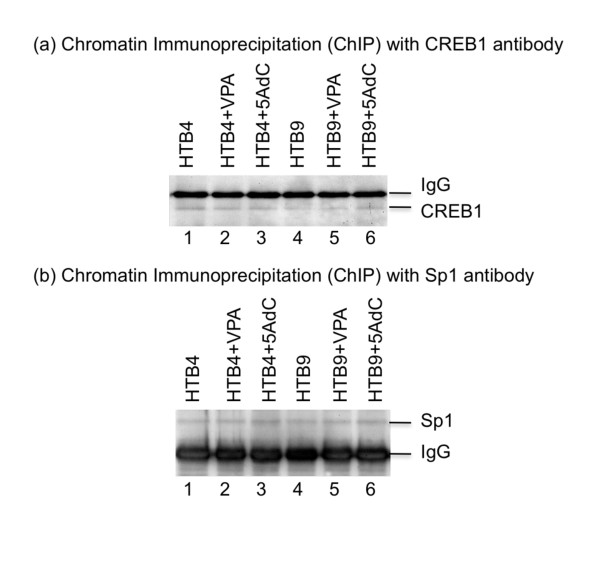
**Detection of CREB-1 and Sp1 protein obtained from the chromatin immunoprecipitation (ChIP)**. A ChIP protocol was performed to pull down the CREB-1 and Sp1 proteins from the individual cell lysates using antibodies against CREB-1 and Sp1 respectively. Half of the agarose beads obtained from the ChIP study were analyzed by western blots to determine the amount of the transcription factors precipitated from individual cell lysates. The remainder of the beads was analyzed by real time PCR to determine the amount of the XPC gene promoter DNA co-precipitated with the individual transcription factors.

To determine the mechanism through which the HDACs inhibit transcription of the XPC gene, we further performed a chromatin immunoprecipitation (ChIP)-based transcription factor binding study. We chose both CREB1 and Sp1 transcription factors for our ChIP study because the consensus sequences for both transcription factors are present at the 5' promoter region of the XPC gene (Figure 1) and are likely to be involved in the transcription regulation of the XPC gene. Some studies also revealed the overlapping in binding to DNA targets between the HDAC4 and the Sp1 [41-46]. The HTB4 and HTB9 cells were treated with the VPA (5 mM) for 48 hours and fixed in 1% formaldehyde. As a control, the untreated HTB4 and HTB9 cells were also harvested and fixed in 1% formaldehyde. The cells were sonicated to shear the chromosomal DNA into small fragments. A ChIP protocol was performed to pull down the CREB1 or the Sp1 transcription factor using antibodies against the individual transcription factors. Half of the beads obtained from the ChIP protocol were analyzed by western blots to determine the amount of the transcription factor pulled down by the ChIP protocol (Figure 2). The remainder of the beads was resuspended into an elution buffer (0.1M Na_2_CO_3_, 1% SDS, 200 mM NaCl) and the DNA co-precipitated with the transcription factors was recovered. The DNA was analyzed by a quantitative PCR (qPCR) protocol to determine the amount of XPC gene promoter region DNA co-precipitated with the transcription factors (Table 3). The results of our western blots revealed that similar amounts of the CREB1 and Sp1 were pulled down from both untreated and VPA-treated cells for both HTB4 and HTB9 cells, suggesting a very successful ChIP protocol (Figure 2). The results of our qPCR studies, however, indicated a very different pattern of XPC gene promoter region DNA co-precipitation following the VPA treatment. When the CREB1 antibody was used in the ChIP study, the VPA treatment resulted in a 4.6 ± 0.4 and 2.2 ± 0.4 fold increase of the co-precipitated XPC gene promoter region DNA in the HTB4 and HTB9 cells respectively (Table 3); when the Sp1 antibody was used in the ChIP study, the VPA treatment caused a 2.2 ± 0.3 and 2.0 ± 0.3 fold increase of the co-precipitated XPC gene promoter region DNA in the HTB4 and HTB9 cells respectively. These results indicate that the VPA treatment enhances the binding of the CREB1 and Sp1 transcription factors at the promoter region of the endogenous XPC gene in both HTB4 and HTB9 cells, suggesting that inhibiting the binding of CREB1 and Sp1 transcription factors to their consensus sequences plays an important role in the HDACs-mediated XPC gene silencing.

**Table 3:** Determination of the level of XPC gene 5' regulatory region DNA co-precipitated with the transcription factors CREB1 and Sp1 by IP in both untreated and VPA-treated HTB4 and HTB9 bladder cancer cells ^*a*^.

IP antigen	HTB4		HTB9	
	
	No treatment	VPA treatment	No treatment	VPA treatment
CREB1	1	4.6 ± 0.4	1	2.2 ± 0.2

Sp1	1	2.2 ± 0.3	1	2.0 ± 0.3

^a ^The level of XPC gene 5' regulatory region DNA co-precipitated in the untreated cells was counted as 1 and the level of XPC gene 5' regulatory region DNA co-precipitated in the VPA-treated cells was calculated as fold change to that of the untreated cells for each cell line. The fold change was expressed as Mean ± S.D. The results were from three independent IP experiments.

### The correlation between the over-expression of HDAC4 and the development of bladder cancer

**Figure 3:**
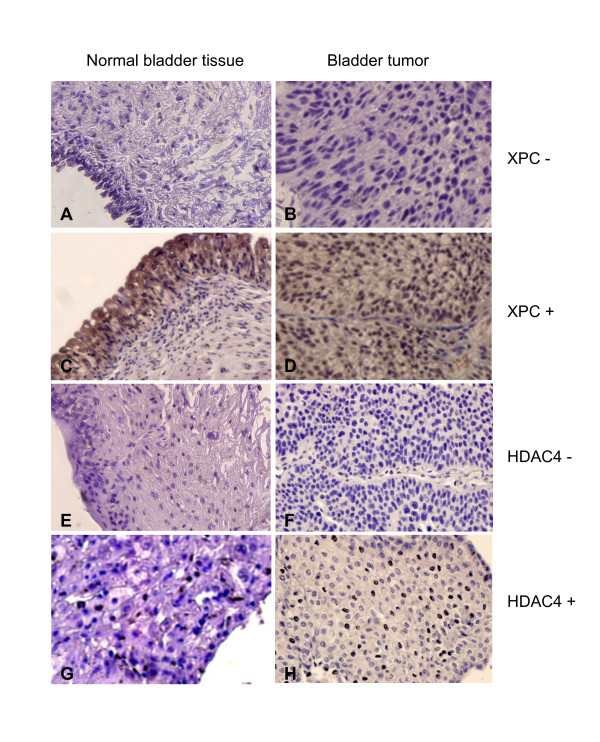
**Immunohistochemistry (IHC) stain of XPC and HDAC4 proteins in both normal and cancerous bladder tissue specimens using bladder tumor tissue arrays**. The bladder tumor tissue arrays purchased from US BioMax Inc. were stained with either XPC or HDAC4 antibodies in an immunohistochemistry (IHC) protocol. The presence of XPC or HDAC4 protein was determined by light microscopy and the image was recorded by a DP Controller software (Olympus Corp., Center Valley, PA).

To further determine the role of HDACs in XPC gene silencing and bladder cancer development, we determined the correlation between the presence of HDACs and the occurrence of bladder cancer using bladder tumor tissue arrays with an immunohistochemistry (IHC) staining procedure (Figure 3 and Table 4). The bladder tumor tissue arrays were purchased from US BioMax, Inc. (Rockville, MD) and used in this study. Both HDAC2 and HDAC4 were chosen for this study because the work of others has revealed abnormal levels of these proteins in many types of cancer [47-55]. The results of our IHC study indicated that the frequency of the HDAC4-positive tissue specimens was much higher in the bladder tumors than in the normal bladder tissues (Figure 3 panel and Table 4). The statistical analysis of the data further revealed a significant difference in the frequency of HDAC4-positive tissue specimens between normal and cancerous bladder tissues (*p *< 0.001) as well as a marginal significance between the increasing incidence of HDAC4 positivity and the increasing severity of the bladder tumors (*p *= 0.08) (Table 4). The frequency of the HDAC2-positive specimens, however, was similar between normal and cancerous bladder tissues (data not shown). These results suggest that over-expression of the HDAC4 is strongly correlated with the development of bladder cancer.

**Table 4:** Determination of the presence of the HDAC4 in both normal and cancerous bladder tissues from bladder tumor tissue arrays.

Type of bladder tissues	# of HDAC4(+)	# of Total tissues	% of HDAC4(+)
Normal bladder tissues	1	23	4.3

Transitional cell carcinomas (Grade 1)	26	58	44.8

Transitional cell carcinomas (Grade 2)	28	59	47.5

Transitional cell carcinomas (Grade 3)	8	25	32.0

*P *value	*p *^Δ ^< 0.001		
	*p*^σ ^= 0.08		

Note: *p *^Δ ^value is the comparison between the group of normal bladder tissues and the group of cancerous bladder tissues. *p*^σ ^is the comparison among the groups of normal bladder tissues, Grade 1, Grade 2, and Grade 3 bladder carcinomas.

### The HDAC4 was over-expressed in most of the bladder cancer cells

**Figure 4:**
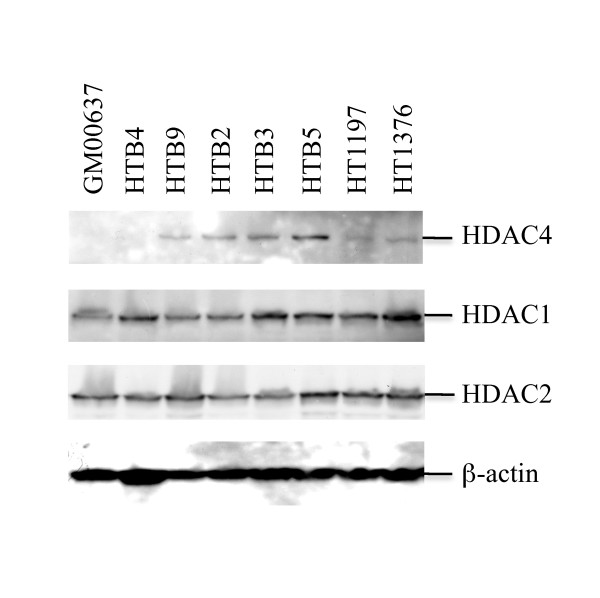
**Detection of expression of HDAC4, HDAC1, and HDAC2 in various bladder cancer cells**. The cell lysates prepared from the HTB2, HTB3, HTB4, HTB5, HTB9, HT1197, HT1376 bladder cancer cells and GM00637 normal human fibroblast cells (30 μg total protein) were analyzed by western blots to determine the protein levels of HDAC4, HDAC1, HDAC2, and β-actin in each cell lysate. The antibodies against HDAC4 (A-4), HDAC1 (C-19), HDAC2 (H-54) and β-actin (C-2) were purchased from Santa Cruz Biotechnology, Inc. (Santa Cruz, CA) and used in the western blots study.

The results of our IHC studies revealed strong correlation between over-expression of the HDAC4 and the occurrence of bladder tumors. To validate the IHC result, we further determined expression of several HDACs, including HDAC4, HDAC1, and HDAC2, in the HTB4, HTB9, HTB2, HTB3, HTB5, HT1197 and HT1376 bladder cancer cells (Figure 4). The expression of these HDACs in the GM00637 normal human fibroblast cells was also determined in the western blotting study and used as a control. The results obtained from our western blots study indicated that the protein levels of the HDAC1 and HDAC2 were similar in all the tested cells (Figure 4 middle panels). In contrast, the expression levels of HDAC4 were greatly increased in most of the tested bladder cancer cells except the HTB4 bladder cancer cells in comparison to that of the GM00637 normal human fibroblast cells (Figure 4 top panel). This result confirmed our IHC results and suggested the important role of HDAC4 over-expression in the bladder cancer development.

### Prior treatment with the HDAC inhibitor VPA enhanced cisplatin-induced apoptosis of bladder cancer cells

**Figure 5:**
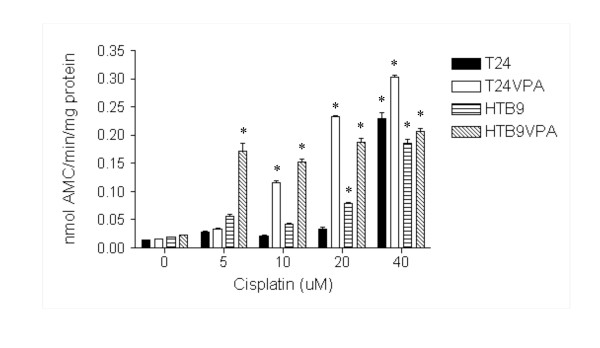
**The cisplatin-induced caspase 3 activity in both untreated and VPA-treated HTB4 and HTB9 bladder cancer cells**. The VPA treatment (5 mM) was done 24 hours prior to the cisplatin treatment. The cells were treated with cisplatin at the indicated concentrations for 3 hours and then cultured in the cell culture incubator for 40 hours before the cells were harvested and the caspase 3 activity was measured. The caspase 3 activity was measured as nanomole of AMC/minute/mg of protein. (* statistical difference to that of the untreated cells with *p *value < 0.01).

Extensive studies have demonstrated the cisplatin-induced apoptosis as major mechanism in cell killing [16,39,56-58]. Because of the important function of XPC protein in the cisplatin-caused apoptosis [16] and the role HDACs in XPC gene silencing, we further investigated the effect of the HDAC inhibitor VPA in cisplatin-induced apoptosis of bladder cancer cells. The HTB4 and HTB9 bladder cancer cells were treated with VPA (5 mM) for 48 hours before they were treated with cisplatin. The cells were harvested 40 hours after the cisplatin treatment and the caspase-3 activity was determined (Figure 5). The caspase-3 activity was also determined from the HBT4 and HTB9 cells that were treated with cisplatin but without the prior VPA treatment (Figure 5). The cisplatin treatment itself caused an increase in caspase-3 activity in both HTB4 and HTB9 bladder cancer cells at high concentrations (20 μM and 40 μM) but not at lower concentrations (5 μM and 10 μM) (Figure 5). When these cells were treated with VPA prior to the cisplatin treatment, however, the caspase-3 activity was significantly increased at lower concentrations as well (Figure 5). For example, when treated only with cisplatin at 10 μM, the caspase 3 activity was increased by a 1.5 and 2 fold in the HTB4 and HTB9 cells respectively; when the cells were treated with 5 mM VPA prior to the cisplatin treatment, however, the 10 μM cisplatin treatment resulted in a 7.3 and 6.6 fold increase of the caspase-3 activity in the HTB4 and HTB9 cells respectively (Figure 5). These results suggest that the prior treatment of HTB4 and HTB9 bladder cancer cells with the HDAC inhibitor VPA sensitizes these bladder cancer cells to the anticancer drug cisplatin.

## Discussion

In this work we have determined the role of HDACs in XPC gene silencing and bladder cancer development. The results obtained from our HDAC inhibitor treatment studies revealed that the VPA treatment led to an increase in transcription of the XPC mRNA in both HTB4 and HTB9 bladder cancer cells. The results obtained from our ChIP study demonstrated that the VPA treatment resulted in an increase in binding of the CREB1 and Sp1 transcription factors at the 5' regulatory region of the XPC gene in both HTB4 and HTB9 cells. The results of our IHC studies further indicated a strong correlation between the over-expression of the HDAC4 and the occurrence of urinary bladder transitional cell carcinomas. In addition, the results obtained from our caspase-3 activation studies also demonstrated that the pre-treatment of HTB4 and HTB9 bladder cancer cells with VPA enhanced the anticancer drug cisplatin-induced activation of caspase-3, an important apoptotic caspase indicative of irreversible apoptosis. Given the important role of the XPC protein in protecting cells against many environmental carcinogen-induced deleterious effects and the significance of the HDACs in epigenetic gene transcription regulation [31-33], these results suggest that the HDACs play an important role in XPC gene silencing and bladder cancer development. Therefore, these results provide an important mechanism of XPC gene silencing and bladder cancer development. Because of the essential role of the XPC protein in initiating DNA damage-induced cellular responses [16], these results further suggest that silencing of the XPC gene may provide a critical early event for initiation of bladder tumors. In addition, the results obtained from these studies further suggests that reactivation of the XPC gene by HDAC inhibitors may have great benefits for bladder cancer treatment, especially for DNA-damaging anticancer drugs such as cisplatin.

The results of our ChIP studies revealed that the VPA treatment led to an increase in binding of the CREB1 and Sp1 transcription factors to the 5' regulatory region of the XPC gene. These results suggest that inhibiting the binding of these transcription factors to their consensus sequences plays an important role in the HDACs-caused XPC gene silencing of bladder cancer cells. This provides an important basis for understanding the mechanism of XPC gene silencing in bladder cancer cells. However, it is widely known that the consensus sequences of many transcription factors are present at the promoter region of the XPC gene, whether or not the bindings of these transcription factors are also affected by HDACs, and therefore, contribute to the XPC gene silencing is largely unknown. It may be important to determine the effect of HDACs on the bindings of these individual transcription factors at the promoter region of the XPC gene in order to provide a better understanding of the molecular basis by which the HDACs cause silencing of the XPC gene in bladder cancer cells.

The results of our IHC studies reveal that the frequency of the HDAC4-positive tissue specimens was significantly increased in the urinary bladder transitional cell carcinomas in comparison to normal bladder tissues. However, the results obtained from our IHC study using a HDAC2 antibody did not show a significant change in the frequency of HDAC2-positive tissue specimens between normal and cancerous bladder tissues (data not shown). Given the similarity between the HDAC2 and HDAC4 proteins in both their functions, these results suggest that only certain HDACs are involved in the XPC gene silencing in the urinary bladder transitional cell carcinomas. Since the HDACs family proteins also include several other HDACs, it would be important to determine the correlation between the presence of the individual HDACs and the bladder cancer occurrence for each HDAC in order to provide a better understanding of the role of specific HDACs in XPC gene silencing and bladder cancer development.

The work described in this study was mainly focused on determining the role of HDACs in XPC gene silencing and bladder cancer development. However, it is known that other epigenetic gene regulation mechanisms, including DNA methylation and microRNA (miRNA), can also lead to silencing of the target genes [32,33]. In fact, recently reported results suggest that DNA methylation may play an important role in XPC gene silencing of lung cancer cells [29]. Therefore, future studies also need to determine the roles of these epigenetic regulation mechanisms in XPC gene silencing and bladder cancer development in order to provide a better understanding of the mechanism of XPC gene silencing and bladder cancer development.

Attenuated XPC protein has been observed in many types of cancer, including bladder and lung cancer [27,59]. Given the strong correlation between environmental carcinogen exposure and cancer occurrence for both bladder and lung cancer as well as the similarity of the lung and bladder organs in exposure to environmental carcinogens, it is possible that silencing of the XPC gene may play an important role in cancer development for many different types of cancer. Therefore, the knowledge obtained from this study will be important not only for understanding the mechanism of bladder cancer development but also for grasping the mechanism of development of these cancers as well. In addition, the knowledge obtained from this study is also important for detection, treatment, and risk assessment of cancer as well as new anticancer drug design and development.

## Competing interests

The authors declare that they have no competing interests.

## Authors' contributions

XX carried out the VPA and IHC studies, and participated in the design and coordination of the project. LW carried out the cell culture and the participated in the immunoblotting and immunoprecipitation study. JA carried out the statistical analysis of the IHC data. GW participated in the design and coordination of the studies and drafted the manuscript. All authors read and approved the final manuscript.
